# Enhancing the Delivery of Erythropoietin and Its Variants into the Ischemic Brain

**DOI:** 10.1100/tsw.2009.104

**Published:** 2009-09-15

**Authors:** Dirk M. Hermann

**Affiliations:** Department of Neurology, University Hospital Essen, Germany

**Keywords:** growth factor, neuroprotection, hematopoietic, biodistribution

## Abstract

The hematopoietic growth factor erythropoietin (EPO) and its neuroprotective, but not hematopoietic, variants asialoEPO, carbamylated EPO (CEPO), and low sialic acid EPO (Neuro-EPO) are attractive candidates for stroke treatment. Due to their large molecular weight, these proteins enter the brain only to a minor extent when intravenously administered, which has raised the question for alternative delivery strategies, among which intranasal delivery may certainly be an attractive choice, as the review by Garcia Rodriguez and Sosa Teste in this journal points out. Before this strategy may be considered clinically applicable, however, more and, in particular, quantitative information is needed about (a) the temporospatial accumulation of EPO and its variants in the brain tissue both in animals and nonhuman primates, and (b) the accumulation of EPO and its variants in the human cerebrospinal fluid.

The blood-brain barrier (BBB) represents a dense monolayer of endothelial cells that are interconnected via tight junctions. Thereby, the BBB efficiently prevents the passage of hydrophilic peptides into the ischemic brain once their molecular weight exceeds a critical size, in the range of 600–1000 Da[1]. Various attempts have been made in recent years to circumvent the BBB, e.g., by gene transfer technologies[2], Trojan horse strategies using lipid vesicles or fusion proteins[1,3], or blockade of BBB efflux transporters[4], so far with limited success that led to clinically applicable strategies.

In their paper just published in *TheScientificWorldJOURNAL*, Garcia Rodriguez and Sosa Teste[5] evaluated the ability of nasal delivery strategies of the hematopoietic growth factor erythropoietin (EPO), as well as of its variants asialoEPO, carbamylated EPO (CEPO), and low sialic acid EPO (Neuro-EPO), to prevent ischemic injury and promote recovery after stroke.

In view of the neuroprotective activity of EPO, considering that this growth factor is already in clinical use in patients with renal failure, it is an attractive candidate for stroke treatment. Successful animal studies[6,7,8] rapidly led to a clinical proof-of-concept trial in which recombinant human EPO reduced ischemic injury and improved functional neurological recovery in acute stroke patients[9]. Following that study, a bigger multicenter trial was conducted that has just been finalized. Existing information again points towards a neuroprotective effect of EPO that is evident when this growth factor is delivered as monotherapy, but not when given in combination with thrombolytics.

That EPO exerts hematopoietic effects besides its neuroprotective activity may pose problems in the treatment of cerebrovascular diseases, as EPO-induced polyglobulia may disturb blood flow, thus compromising the survival of the tissue[10]. Variants of EPO that do not bind to the classical EPO

receptor may overcome this issue, as these compounds exert neuroprotective, but not hematopoietic, actions. These variants that include asialoEPO, CEPO, and Neuro-EPO indeed promote survival of ischemic tissue in rodents[11], and they may also be used for intranasal delivery, as the authors point out.

Yet, does the successful nasal delivery in rodents imply that this strategy can also be translated to human patients? Human patients differ from rodents in regards to the size of their brain and olfactory bulb, which are much bigger in humans than rodents. Even if, as the authors point out, intranasally delivered EPO enters the brain within minutes in rats, mice, or gerbils, this does not necessarily translate to human patients. Importantly, the authors provide the first experimental evidence that recombinant human EPO becomes detectable in the cerebrospinal fluid of monkeys within only a few minutes after nasal delivery[12]. Yet, whether the EPO also reaches brain areas at risk and whether the tissue levels achieved are sufficient for protecting the tissue remains to be shown.

In view of its transport along the olfactory nerve, EPO is supposed to accumulate in projection areas of the olfactory bulb first. From these areas, the molecule still needs to pass on to ischemic regions, which are at a far distance from these projection structures that are mainly located in the basal forebrain. Hence, the accumulation of EPO in ischemic tissue may need considerably longer than that in the CSF. As such, nasal delivery remains experimental at this stage, and additional data that look at the accumulation behavior in various brain structures are needed to provide additional proofs of concept.

It is noteworthy that modified EPO derivatives, namely low sialic acid Neuro-EPO, can also be applied via the nasal route. However, will these derivatives be finally successful in the treatment of human stroke? A big advantage is that the lack of hematopoietic activity would open new opportunities to deliver EPO over longer intervals, e.g., in the postacute phase of the stroke. In that case, polyglobulia induced by EPO would be a relevant concern in the clinical setting. Besides its hematopoietic effect, recombinant human EPO also causes alterations in platelet function and hemostasis, which may result in potential complications. The neuroprotective derivatives of EPO probably lack this action, as shown for CEPO in the past[13]. This makes these molecules interesting candidates for stroke treatment.

It should be kept in mind, however, that classical EPO also exerts stabilizing effects on blood vessels, which may contribute to the survival-promoting effects of the growth factor in human patients. As such, EPO enhances blood flow, reduces BBB leakage, and promotes angiogenesis[14,15]. That these non-neuronal effects, which CEPO and other derivatives presumably do not share, are part of EPO’s rescue effects after systemic intravenous delivery in patients is very likely, particularly as this growth factor probably passes the BBB only to a minor extent. It has been shown that EPO accumulates in the brain after systemic delivery[6]. The dynamics and degree of the BBB passage, however, are poorly known. As such, open questions remain that need to be resolved before EPO's variants should enter clinics.
